# Lessons from the Field: Integrated survey methodologies for neglected tropical diseases

**DOI:** 10.1093/trstmh/traa132

**Published:** 2020-12-10

**Authors:** Emma M Harding-Esch, Molly A Brady, Cristiam A Carey Angeles, Fiona M Fleming, Diana L Martin, Scott McPherson, Hollman Miller Hurtado, John M Nesemann, Benjamin C Nwobi, Ronaldo G C Scholte, Fasihah Taleo, Sandra Liliana Talero, Anthony W Solomon, Martha Idalí Saboyá-Díaz

**Affiliations:** Faculty of Infectious and Tropical Diseases, Clinical Research Department, London School of Hygiene & Tropical Medicine, London, WC1E 7HT, UK; Global Health Division, RTI International, Washington, D.C. 20008, USA; Pan American Health Organization, Iquitos, 16000, Perú; SCI Foundation, London, SE11 5DP, UK; Division of Parasitic Diseases and Malaria, US Centers for Disease Control and Prevention, Atlanta GA 30030, USA; Global Health Division, RTI International, Washington, D.C. 20008, USA; Department of Vector Borne and Neglected Infectious Diseases, Vaupés Health Directorate, Mitú, 970001 Vaupés, Colombia; Francis I Proctor Foundation, San Francisco, 94122, USA; Global Health Division, RTI International, Washington, D.C. 20008, USA; Neglected, Tropical, and Vector-Borne Diseases Unit, Communicable Diseases and Environmental Determinants of Health Department, Pan American Health Organization, Washington, D.C. 20037, USA; Department of Neglected Tropical Diseases, Vanuatu Ministry of Health and Medical Services, Port Vila, Vanuatu; Research and Innovation Department, Superior School of Ophthalmology, Barraquer Institute of America, 110221 Bogotá, Colombia; Department of Control of Neglected Tropical Diseases, World Health Organization, Geneva 1211, Switzerland; Neglected, Tropical, and Vector-Borne Diseases Unit, Communicable Diseases and Environmental Determinants of Health Department, Pan American Health Organization, Washington, D.C. 20037, USA

**Keywords:** cost-effectiveness, integrated, neglected tropical diseases, surveys

## Abstract

The 2021–2030 Neglected Tropical Diseases road map calls for intensified cross-cutting approaches. By moving away from vertical programming, the integration of platforms and intervention delivery aims to improve efficiency, cost-effectiveness and programme coverage. Drawing on the direct experiences of the authors, this article outlines key elements for successful integrated surveys, the challenges encountered, as well as future opportunities and threats to such surveys. There are multiple advantages. Careful planning should ensure that integration does not result in a process that is less efficient, more expensive or that generates data driving less reliable decisions than conducting multiple disease-specific surveys.

## Introduction

The 2021–2030 road map for neglected tropical diseases (NTDs) sets out new and renewed targets to be achieved through activities that intersect multiple diseases. Three pillars are invoked: accelerating programmatic action; intensifying cross-cutting approaches; and country ownership.^1^ It also recognises the need for more radical change to integrate mainstream approaches within national health systems and coordinate actions across sectors. To monitor and evaluate progress towards NTD road map targets, integrated surveys are a logical tool. Their use would align with the proposed shift to cross-cutting approaches, structures and ways of working. In this paper, we reflect on our experiences to outline key elements for success in conducting integrated NTDs surveys, the challenges encountered and opportunities to expand their use to help achieve NTD goals.

## What are the keys to successful integrated surveys?

Careful planning is key (Box 1). Ultimately, success will depend on national government buy-in and leadership at all levels for each stage of the survey process, protocol design, implementation, data analysis and interpretation. National programme managers should work with key stakeholders and disease experts to create a set of clear objectives that respond to the needs of each disease or disease group, and comply with international recommendations and standardised guidelines. Competing priorities are likely to arise and require discussion. Local capacity and knowledge-sharing for ownership should be developed and/or strengthened. Once a protocol is agreed, all necessary governmental approvals should be put in place. Procurement processes should limit the duplication of items and wasted expenditure, and the logistics for all fieldwork activities should be planned in detail.

Box 1. **Key elements for successful integrated surveys**

**Planning**

- All key stakeholders, competing priorities and mitigation strategies identified.
- National government buy-in and leadership at all levels and for each stage of the survey process.
- Support of national and international experts.
- Protocol developed with input from experts for all diseases included.
- Clear survey objectives.
- Development and/or strengthening of local capacity and knowledge-sharing.
- All correct equipment planned and procured.
- Well-planned logistics for all fieldwork activities.
- Sufficient time and budget allocated for the survey.
- All necessary approvals in place.

**Training**

- Sufficient planning, coordination and funding for training.
- National and local programme managers involved in the selection, training and supervision of field team members.
- Standardised training materials.
- Training programme combines theory with field practice.
- Means to assess survey task competence.
- Specific roles and responsibilities assigned to each individual.
- Field team member movements and sample handling reviewed in detail.
- Fieldwork pilot.

**Implementation**

- Good community awareness and mobilisation.
- Strong supervision.
- Team leads who are technical experts in the different diseases.
- Organise and guarantee logistics for storage and transportation of samples according to the local context, capacity and survey needs.
- Adequate selection of point-of-care (POC) diagnostic tests, well-established procedures to handle multiple POC diagnostic tests and take advantage of multi-disease diagnostic platforms.
- Limit questionnaires to the minimum data needed for programmatic decision-making.
- Electronic data capture and management.
- Automated data analyses.

Well-prepared and comprehensive training is also critical. Strict compliance with the survey protocol is needed to guarantee that all programmes involved receive high quality data and that the conduct of fieldwork is ethical. Standardised training (contents, materials, trainers, etc.) with clear trainee evaluation parameters is key to success. The involvement of national and local programme managers in the selection, training and supervision of field team members is also critical. Integrated surveys demand training of field teams on fieldwork aspects for all the diseases being surveyed (e.g. technical, technological, biosafety, ethical, communications, cultural, work flows, responsibilities). This should always include in-classroom and field sessions that will help to identify challenges to be solved before actual survey fieldwork begins. Integrated surveys usually need more days to train field team members. A particular aspect to check during the training period is the development of an optimal order for examination and/or sample collection for the various diseases of interest. Sufficient planning, coordination and funding for training should be in place.

To successfully implement integrated fieldwork, it is important for communities to be involved from planning through to feedback of results, to increase the acceptability of surveys and any interventions implemented based on them. Electronic data capture and management helps to ensure streamlined, high-fidelity data collection, and automated analysis reduces the time lag to results to inform programmatic decision-making. Strong supervision from the outset of the fieldwork helps to identify and mitigate problems in time to allow for course correction.

## What are the challenges to conducting integrated surveys?

Integrated survey design might be complex if experts from the different disciplines, diseases and hierarchical levels are not involved. It can be a challenge to reach consensus on the survey questions, as well as on parameters such as sample size, age groups, sampling approach, sampling location (school, community, sentinel sites), seasonality, diagnostics, data capture systems, analytical procedures and timelines for delivery of analyses. If differences of opinion for choosing these parameters are not reconciled, this can result in compromises in the epidemiological robustness of data for one or more diseases. Although it could be argued that the survey methodology should be based on the question that requires the greatest epidemiological rigour, disease-specific elimination targets (and funding) mean compromise may not be an option and disease-specific surveys remain the mainstay.

Economic analyses may help to determine whether integrated surveys are cost-effective for a specific disease when additional field elements are required to enable integration. For example, trachoma and most skin NTDs can be diagnosed clinically by well-trained health workers, whereas NTDs that require sample collection and processing can be more time-consuming. Sociocultural factors must also be considered, for example, questions about hydrocele should be asked by someone of the appropriate gender and with consideration for privacy, which may increase the survey time and costs. The choice of diseases to integrate must therefore be made carefully.

## What are the opportunities for and threats to integrated surveys? 

The 2021–2030 NTD road map's proposal for more coordinated action should increase political will for integrated approaches. That will allow national health systems to encourage the cross-partner and multi-sectoral coordination needed for integration. Otherwise, integration can be difficult, due to different sources of funding being ear-marked for specific activities and with different reporting timelines, particularly if responsibility for implementation lies in different health ministry departments. The greatest threat to integrated surveys may in fact be fear of loss of control, power or funding.

The road map's call for coordination could be used to review existing NTD indicators and survey methods to determine overlap and opportunities for integration. One opportunity is a standardised survey process that has a high standard of quality assurance and quality control for all diseases, and a single platform for electronic data capture and management, which can be adapted to incorporate new survey types such as, for example, a modular, multi-disease instrument. The Global Trachoma Mapping Project and Tropical Data (https://www.tropicaldata.org/) have demonstrated that such a system can be successfully deployed globally, at scale.^2^ There is, however, a need for standardised guidelines and protocols for integrated surveys, as well as agreement on which data variables to collect.^3^ Furthermore, integration necessitates continuity of investment in methods, skilled personnel, materials and infrastructure, to sustain national, institutional and community ownership and engagement.

The suggested next steps include conducting integrated surveys using methods with which, and/or locations in which, success seems likely. Surveys in refugee camps or remote populations could be a good test case. Additionally, non-invasive knowledge, attitudes and practice questions can be added to questionnaires in an existing disease-specific survey to provide preliminary data on other diseases. In dispersed and remote populations, integrated surveys have provided an opportunity to collect updated sociodemographic and health data, which in turn has enabled interventions to be implemented to address factors associated with the presence of NTDs. Integrated surveys also offer an opportunity for communities and health workers to learn about diseases with which they were previously unfamiliar, including their prevention, diagnosis, sequelae and management. As more integrated surveys are conducted, the benefits accrued (especially in remote communities or settings with high implementation costs) can enable further funding to be leveraged, and the experience and expertise gained can help to refine methodologies and identify gaps in knowledge and resources.

Another key opportunity for integrated surveys is the availability of laboratory technologies to obtain data for several diseases from a single sample, such as multiplex serological assays.^4^ NTD programmes have already piloted integrated serosurveys and have the opportunity to demonstrate benefits through collaborations that design, implement, analyse and use results for decision-making for several diseases. Serosurveys also provide NTD programmes with an opportunity to trailblaze inter-disease cooperation, partnering with programmes beyond NTDs, such as TB, malaria, HIV or the Expanded Programme on Immunisation, ultimately resulting in greater community benefits. Geospatial modelling^5^ provides an opportunity to determine whether the NTD surveillance data obtained within the platforms used by these non-NTD programmes would be sufficiently epidemiologically rigorous for estimating NTD prevalence.

### Conclusions

There are multiple advantages to integrated surveys for NTDs, and a myriad of opportunities for future refinement and improvement. Careful planning should ensure that integration does not result in a process that is less efficient, more expensive, or which generates data driving less reliable decisions than conducting multiple disease-specific surveys.

## Data Availability

None.
